# Structure of the fungal community in the agricultural system in upland and native area Cerrado

**DOI:** 10.1186/1753-6561-8-S4-P130

**Published:** 2014-10-01

**Authors:** Elainy Cristina Alves Martins Oliveira, Alana de Almeida Valadares, Acacio Aparecido Navarrete, Siu Mui Tsai, Waldesse Piragé de Oliveira Junior, Paula Benevides de Morais

**Affiliations:** 1 Universidade Federal do Tocantins, Palmas, Brazil; 2 CENA - USP, Piracicaba, Brazil

## 

The Cerrado is the second largest biome in the country by area, located mainly in the central highlands of Brazil. Few details about the effect of managements in Cerrado soils that characterize the specific function of this soil biota in sustainable development of their fertility. Even with the lack of information on species of soil fungi in the Cerrado, there are few studies aimed to contribute to the knowledge related to fungi in this biome. The aim of this study was to evaluate the effect of agriculture tecnificada in fungal community structure of soil, using independent techniques of cultivation, as well as compare the structural similarity of the fungal community present in an agricultural soil and an area of native Cerrado. The 20 soil samples were collected in an area of high land (municipality of Paraíso - TO), at the beginning of planting (dec/2012) and next harvest of soybean (feb/2013). For DNA extraction, we used the *Kit Power Soil DNA Extraction™*(MoBio Laboratories, Carlsbad, CA). Reactions PCR/ARISA were prepared to a final volume of 25 μL according Sequerra at al. 1997. The ARISA technique was made into a sequencer ABI PRISM 3100 Genetic Analyzer (Applied Biosystems). To check the electropherograms was used PeakScanner program version 1.0 (Applied Biosystems, Foster City, CA). The quantification using the NanoDrop ^® ^2000 Thermo Scientific, showed that extraction was successful and the DNA's were in good quality. In a visual analysis of the electropherograms, we observed a clear difference in the structure of the fungal community in the agricultural environment and the native area. It is possible to note the presence of peaks that appear in the electropherogram of an environment and that are not present in the other, reflecting AFL's unique in environment. In the analysis of the wealth of AFL's, fungal community showed greater wealth in the native environment, this area also showed the highest number of AFL's unique. The ANOSIM analysis showed that, in general, the fungal community is differed from the agricultural and native environment in both samplings. Thus, this environments studied are characterized by an electropherogram, showing the potential of the ARISA technique to discriminate community structure soil fungi. The observed divergences in fungal community structure reflect differences in the environments evaluated, often due to the physico-chemical differences of each soil.
